# Construction and validation of explainable machine learning models to predict in-hospital mortality for patients with acute type A aortic dissection surgery

**DOI:** 10.3389/fmed.2026.1872110

**Published:** 2026-08-26

**Authors:** Keyan Liu, Sili Shan, Haolong Zeng, Bo Li, Zhicheng Zhu, Jiangbin Sun, Xuezheng Wang, Huiyan Sun, Cuilin Zhu, Kexiang Liu

**Affiliations:** 1 Department of Cardiovascular Surgery, The Second Hospital of Jilin University, Changchun, Jilin, China; 2 School of Artificial Intelligence, Jilin University, Changchun, China; 3 Guilin People’s Hospital, Guilin, Guangxi, China

**Keywords:** acute type A aortic dissection, machine learning, mortality risk, predictive analytics, prognostic factors, random forest

## Abstract

**Objective:**

To construct and validate a risk prediction model of in-hospital mortality using machine learning (ML) algorithm in a retrospective cohort of acute type A aortic dissection (ATAAD) patients undergoing surgical treatment.

**Methods:**

Patients with ATAAD undergoing surgical treatment between January 2014 and December 2022 were enrolled to predict in-hospital mortality. To address class imbalance and overfitting, we developed a robust Random Forest (RF)-based classification framework using a nested stratified 5-fold cross-validation (NCV). This was a single-center, retrospective study with internal validation only; no external validation was performed. Performance was evaluated via ROC-AUC, Precision-Recall Area Under the Curve (PR-AUC), sensitivity, brier score and calibration metrics, with Shapley Additive exPlanations (SHAP) utilized for feature interpretation.

**Results:**

A total of 639 ATAAD patients were included in the analytical cohort, with an in-hospital mortality rate of 5.6% (36/639). The calibrated full RF model (50 preoperative clinical variables) achieved an ROC-AUC of 0.666, PR-AUC of 0.145, brier score of 0.051, and calibration slope of 0.836, with a sensitivity of 0.694 at an optimized threshold. A parsimonious 15-feature model maintained robust performance (ROC-AUC: 0.752, PR-AUC: 0.207, brier score: 0.050 and calibration slope: 0.934). SHAP analysis identified Creatine Kinase-MB, Myoglobin, and Fibrinogen Concentration as the top mortality predictors.

**Conclusion:**

We developed and internally validated an explainable RF model to predict in-hospital mortality after ATAAD surgery. Given the low positive predictive value and high negative predictive value, the model is best regarded as a promising preliminary rule-out/triage tool that requires multicenter external validation before clinical use.

## Introduction

Acute type A aortic dissection (ATAAD) is a severe condition and the mortality rate increases by 1% and 2% per hour, eventually reaching up to 60% (1). Despite the improvement in medical management and surgical technique, the in-hospital mortality in patients with ATAAD remains high, as reported to be 7.1–18.4% after surgical repair of ATAAD (2). Multiple risk factors have been identified to be involved in the ATAAD surgery, including advanced age, preoperative severe conditions, malperfusion syndrome, and renal failure were independent risk factors of postoperative mortality in ATAAD patients (3). The European System for Cardiac Operative Risk Evaluation II (EuroSCORE II) is currently a widely used scoring system for cardiovascular surgery, but it is not specifically designed for ATAAD surgery (4). Accurate prediction models for cardiovascular procedures are critical for patient selection, risk stratification, tailoring therapy, and determining prognosis. Consequently, it is critical to develop an accurate scoring system to predict the perioperative risk for patients undergoing ATAAD surgery.

In recent years, machine learning (ML) algorithms have been increasingly employed to develop prediction models in clinical medicine, demonstrating superior predictive performance compared to traditional approaches. ML methodologies have the potential to unravel intricate patterns by leveraging advanced algorithms and computational techniques, thereby empowering clinicians with actionable insights for personalized patient care (5). Particularly in cohorts that include non-linear variables, ML techniques outperform conventional statistical models in predicting outcomes across various settings (6). In clinical medical data, the presence of missing data due to incomplete sampling, the imbalance between experimental and control group samples size, and the necessity for clinical predictions to be highly interpretable pose significant challenges for the application of ML. Especially, the application of ML techniques to predict in-hospital mortality following ATAAD surgery is still lacking.

In this study, we aimed to develop and validate a Random Forest (RF)-based ML framework for predicting in-hospital mortality among patients undergoing surgery for ATAAD. Additionally, in the context of imbalanced dataset of surgical mortality, various techniques and evaluation tests were employed to further optimize the predictive efficacy of the ML algorithms.

## Materials and methods

### Patient enrollment

This retrospective, single-center study included adult patients who underwent surgical repair for ATAAD at our institution between January 2014 and December 2022. Patients diagnosed with chronic type A aortic dissection were excluded at the outset. Of the 677 source patients, 38 (5.61%) were excluded because all preoperative biochemical laboratory values were missing, leaving a final analytical cohort of 639 patients (603 survivors and 36 non-survivors). Review of the raw data showed that 31 of these exclusions occurred in 2018, whereas only 2, 2, 1, and 2 occurred in 2019–2022, respectively, suggesting that the missingness was concentrated in the early study period and likely reflected systematic early-record limitations rather than data missing completely at random.

By design, the intended prediction time was after the initial admission assessment but before surgery. Only variables available at that time point were eligible. Intraoperative and postoperative variables—including circulatory-arrest duration, cerebral-perfusion strategy, and the type/extent of surgical repair—were intentionally excluded so that the model uses no information arising after the prediction time. This is a deliberate design decision to build a presurgical triage tool, not a data limitation.

### Overall framework

**FIGURE 1:**
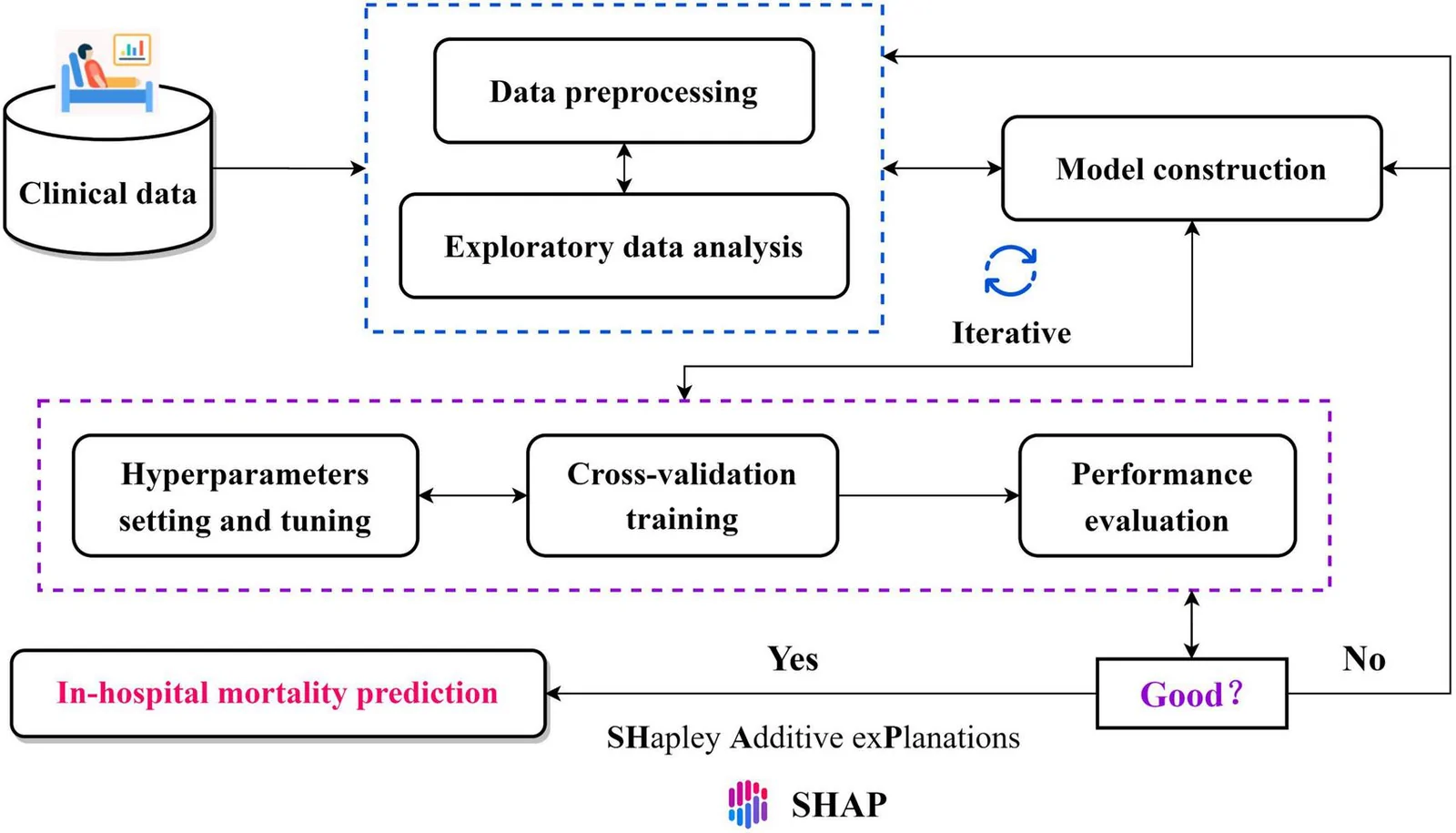
The overall framework of this study. It outlined the iterative development process of a data-driven predictive model for in-hospital mortality.

In this study, we propose an integrated ML framework to predict in-hospital mortality (Figure 1). It takes the prediction as a binary classification problem (7), that is survival or death. This pipeline is specifically designed to address common clinical research challenges, including limited sample sizes, missing data, and severe class imbalance.

Following exploratory data analysis (EDA), we utilized a RF classifier integrated within a nested stratified 5-fold cross-validation (NCV) framework (8, 9). Furthermore, hyperparameter optimization is conducted based on Bayesian theory (10). To prevent data leakage, fold-wise multivariable imputation was implemented. Within the inner loop, hyperparameter tuning was executed via Optuna (11), specifically targeting the maximization of the Precision-Recall Area Under the Curve (PR-AUC) to optimally handle label imbalance. Furthermore, the framework incorporated *post hoc* probability calibration and dynamic threshold optimization to address data heterogeneity.

Based on this robust training pipeline, we evaluated two prespecified models: a full model incorporating all 50 eligible preoperative variables, and a parsimonious model restricted to the top 15 features ranked by average feature importance derived from the full model. For the parsimonious model, the top 15 features were selected within each outer-training fold according to the mean impurity-based feature importance from the full-variable RF model. The corresponding held-out outer validation fold was not used for feature ranking or feature selection. Two variables were excluded *a priori* from the 52 available fields—estimated glomerular filtration rate (a value derived from creatinine) and high-sensitivity troponin (missing in 60.9% of patients)—leaving 50 predictors. This dual-model strategy was adopted to compare maximal predictive complexity against practical bedside deploy ability while mitigating overfitting.

Finally, predictive performance was comprehensively assessed using the Area Under the ROC Curve (AUC), PR-AUC, Sensitivity, Specificity, Brier score, and calibration metrics, explicitly avoiding overall accuracy due to its misleading nature in imbalanced scenarios. To ensure clinical transparency, SHapley Additive exPlanations (SHAP) were employed to elucidate decision-making processes and quantify the significance of individual predictors (12).

### Data collection and preprocessing

Data preprocessing and EDA were conducted in a Python 3.8.10 environment (13), primarily utilizing the Pandas, NumPy, Matplotlib, missingno, and Sweetviz libraries. Specifically, the Sweetviz tool was employed to generate information-dense visualizations and correlation analyses via a self-contained web application, facilitating a comprehensive initial EDA. Concurrently, the missingno package provided visual summaries of missing data patterns to assess dataset completeness. For the further development of the RF prediction model, categorical variables were encoded based on their characteristics: patient sex was assigned values of 1 and 2 (1 = male, 2 = female), while binary features (e.g., medical history and symptoms) were encoded as 0 or 1 (0 = no, 1 = yes). The primary clinical endpoint was defined as in-hospital mortality (all-cause death during hospitalization).

Following initial formatting, we evaluated data completeness and per-variable missingness rates are provided in Table 1. To address missing values, we employed the Iterative Imputer with a fixed random seed and a maximum of 10 iterations. To prevent data leakage, Missing values were handled with an Iterative Imputer fitted only on the predictor matrix of each outer-training fold and applied to the corresponding validation fold; outcome labels were held separately and were never provided to the imputer, thereby preventing outcome leakage. Alternative-imputation and complete-case sensitivity analyses were not performed in the present study and are proposed as future work.

**TABLE 1:** The initial laboratory test results on admission.

Variable name	Unit	Summary measure	Valid clinical range	Missing N (%)	Survivors (*N* = 603)	Non-survivors (*N* = 36)	*P*-value
D-Dimer	mg/L	Median [Q1, Q3]	0–0.50	4 (0.6%)	5.86 [2.36, 12.88]	9.82 [4.86, 22.55]	0.0121*
eGFR	mL/min	Median [Q1, Q3]	>90	31 (4.9%)	85.60 [55.60, 101.50]	76.90 [48.60, 95.60]	0.1851
γ–glutamyl transpeptidase	U/L	Median [Q1, Q3]	10.0–60.0	0 (0.0%)	32.00 [20.00, 62.50]	36.50 [22.75, 64.00]	0.5895
Albumin	g/L	Median [Q1, Q3]	40.0–55.0	0 (0.0%)	40.20 [37.50, 42.80]	39.00 [35.77, 41.17]	0.0783
White blood cell count	10^∧^9/L	Median [Q1, Q3]	3.5–9.5	0 (0.0%)	11.40 [9.20, 14.10]	12.15 [10.18, 15.65]	0.1340
Alanine aminotransferase	U/L	Median [Q1, Q3]	7–40	0 (0.0%)	22.00 [14.00, 36.50]	24.00 [17.75, 40.25]	0.1130
Aspartate aminotransferase	U/L	Median [Q1, Q3]	13–35	0 (0.0%)	22.00 [17.00, 37.00]	35.50 [21.00, 76.75]	0.0077*
Red blood cell count	10^∧^12/L	(Mean ± SD)	3.80–5.10	0 (0.0%)	4.34 ± 0.58	4.27 ± 0.62	0.5473
Creatinine	μmol/L	Median [Q1, Q3]	41–81	0 (0.0%)	83.00 [65.00, 116.00]	85.50 [63.50, 118.75]	0.7875
Alkaline phosphatase	U/L	Median [Q1, Q3]	45–125	0 (0.0%)	73.00 [58.00, 90.00]	76.00 [63.50, 92.50]	0.2574
Blood urea nitrogen	mmol/L	Median [Q1, Q3]	3.10–8.80	0 (0.0%)	6.82 [5.44, 8.86]	7.09 [6.18, 9.18]	0.3044
Uric acid	μmol/L	Median [Q1, Q3]	89–375	0 (0.0%)	377.00 [296.00, 465.50]	373.00 [325.00, 437.00]	0.9741
Hemoglobin content	g/L	Median [Q1, Q3]	115–150	0 (0.0%)	134.00 [122.00, 147.00]	132.00 [125.00, 138.25]	0.5660
Platelet count	10^∧^9/L	Median [Q1, Q3]	125–350	0 (0.0%)	169.00 [134.50, 210.50]	150.50 [124.00, 198.00]	0.1640
Platelet-to-lymphocyte ratio	%	Median [Q1, Q3]	50–150	3 (0.5%)	160.31 [108.94, 227.27]	168.04 [122.92, 252.44]	0.4819
Direct bilirubin	μ/L	Median [Q1, Q3]	0.00–8.00	0 (0.0%)	5.25 [3.80, 7.35]	4.89 [3.65, 6.93]	0.5201
Total bilirubin	μ/L	Median [Q1, Q3]	0.00–23.00	0 (0.0%)	17.50 [12.89, 23.65]	15.45 [11.88, 20.65]	0.2945
Total protein	g/L	Median [Q1, Q3]	65.0–85.0	0 (0.0%)	65.10 [61.20, 68.80]	63.45 [58.85, 68.58]	0.2031
Partial thromboplastin time	s	Median [Q1, Q3]	25.1–36.5	5 (0.8%)	31.50 [29.35, 33.70]	32.20 [28.65, 34.00]	0.9621
Monocyte count	10^∧^9/L	Median [Q1, Q3]	0.1–0.6	1 (0.2%)	0.80 [0.60, 1.00]	0.70 [0.47, 1.00]	0.4178
High-sensitivity troponin	pg/mL	Median [Q1, Q3]	0–17.5	389 (60.9%)	33.70 [8.25, 231.95]	75.40 [11.50, 7102.40]	0.2271
Myoglobin	ng/mL	Median [Q1, Q3]	17.40–105.7	7 (1.1%)	46.45 [23.58, 121.25]	104.00 [29.43, 865.48]	0.0046*
Creatine kinase-MB	ng/mL	Median [Q1, Q3]	0.600–6.300	7 (1.1%)	2.00 [1.10, 5.15]	5.55 [1.50, 62.05]	0.0018*
Potassium	mmol/L	Median [Q1, Q3]	3.50–5.30	0 (0.0%)	3.82 [3.54, 4.12]	4.01 [3.48, 4.35]	0.1242
Lymphocyte count	10^∧^9/L	Median [Q1, Q3]	1.1–3.2	1 (0.2%)	1.00 [0.70, 1.50]	0.90 [0.67, 1.30]	0.1116
Sodium	mmol/L	Median [Q1, Q3]	137.0–147.0	0 (0.0%)	139.50 [137.60, 141.50]	140.30 [137.88, 141.78]	0.3500
International normalized ratio	/	Median [Q1, Q3]	0.80–1.20	5 (0.8%)	1.05 [0.99, 1.12]	1.10 [1.04, 1.21]	0.0120*
Prothrombin time	s	Median [Q1, Q3]	9.4–12.5	5 (0.8%)	12.30 [11.60, 13.10]	12.90 [12.10, 14.20]	0.0114*
Mean platelet volume	fl	Median [Q1, Q3]	7.0–11.0	1 (0.2%)	10.20 [9.50, 10.90]	10.25 [9.47, 10.90]	0.9610
Basophil percentage	%	Median [Q1, Q3]	0.0–1.0	1 (0.2%)	0.20 [0.10, 0.30]	0.20 [0.10, 0.20]	0.2638
Basophil count	10^∧^9/L	Median [Q1, Q3]	0–0.06	1 (0.2%)	0.02 [0.01, 0.03]	0.02 [0.01, 0.03]	0.6630
Eosinophil percentage	%	Median [Q1, Q3]	0.4–8	1 (0.2%)	0.10 [0.00, 0.30]	0.00 [0.00, 0.10]	0.0048*
Eosinophil count	10^∧^9/L	Median [Q1, Q3]	0.02–0.52	1 (0.2%)	0.00 [0.00, 0.03]	0.00 [0.00, 0.01]	0.0227*
Fibrinogen degradation products	μg/mL	Median [Q1, Q3]	0–5.00	4 (0.6%)	20.60 [8.78, 45.55]	33.30 [16.70, 85.20]	0.0107*
Fibrinogen measurement	g/L	Median [Q1, Q3]	2.00–4.00	5 (0.8%)	2.79 [2.08, 3.88]	2.11 [1.41, 3.12]	0.0032*
Neutrophil-to-lymphocyte ratio	%	Median [Q1, Q3]	1.0–3.0	1 (0.2%)	9.67 [5.55, 14.62]	12.54 [7.62, 16.54]	0.0293*
Neutrophil count	10^∧^9/L	Median [Q1, Q3]	1.8–6.3	1 (0.2%)	9.51 [7.17, 12.04]	10.49 [8.72, 13.80]	0.0541

*Indicates that the *p*-value is significant.

### Model construction

To predict in-hospital mortality, we developed a calibrated, multi-stage pipeline centered on a RF classifier (14). With only 36 in-hospital deaths, the descriptive events-per-variable (EPV) ratio was 36/50 = 0.72 for the full model and 36/15 = 2.40 for the parsimonious model. Both values are low and indicate a real risk of overfitting, particularly for the full model; nested cross-validation reduces optimism from hyperparameter tuning but cannot eliminate instability caused by sparse events. Against this background, RF was selected because it represents non-linear effects and variable interactions, requires no predictor scaling, and integrates naturally with fold-wise imputation, probability calibration, and SHAP interpretation. However, our choice was based on methodological suitability rather than demonstrated superiority. Class imbalance was handled through stratified cross-validation, PR-AUC as the inner-loop optimization target, and *post hoc* threshold selection. We deliberately did not apply class weighting or synthetic oversampling, so as to avoid distorting the underlying risk distribution before isotonic calibration. Accordingly, class imbalance was addressed using this metric-driven, threshold-moving strategy rather than through class weighting or synthetic oversampling.

Furthermore, we integrated a *post hoc* calibration layer using Isotonic Regression to refine raw ensemble outputs into actionable clinical risk probabilities. This refinement is essential to correct the inherent tendency of RF models to yield distorted or overconfident probability distributions. By mapping raw scores to well-calibrated posterior probabilities, we ensured that predicted risks were robustly aligned with observed clinical outcomes, facilitating more reliable early risk stratification and clinical triage.

In addition to the full-variable model, a parsimonious model was also developed. Candidate variables for the parsimonious model were selected according to the mean feature importance derived from the full model, and the reduced predictor set was then entered into the same modeling framework. All analyses were conducted in Python using the scikit-learn and Optuna libraries (15).

### Model training and hyperparameters tuning

**FIGURE 2:**
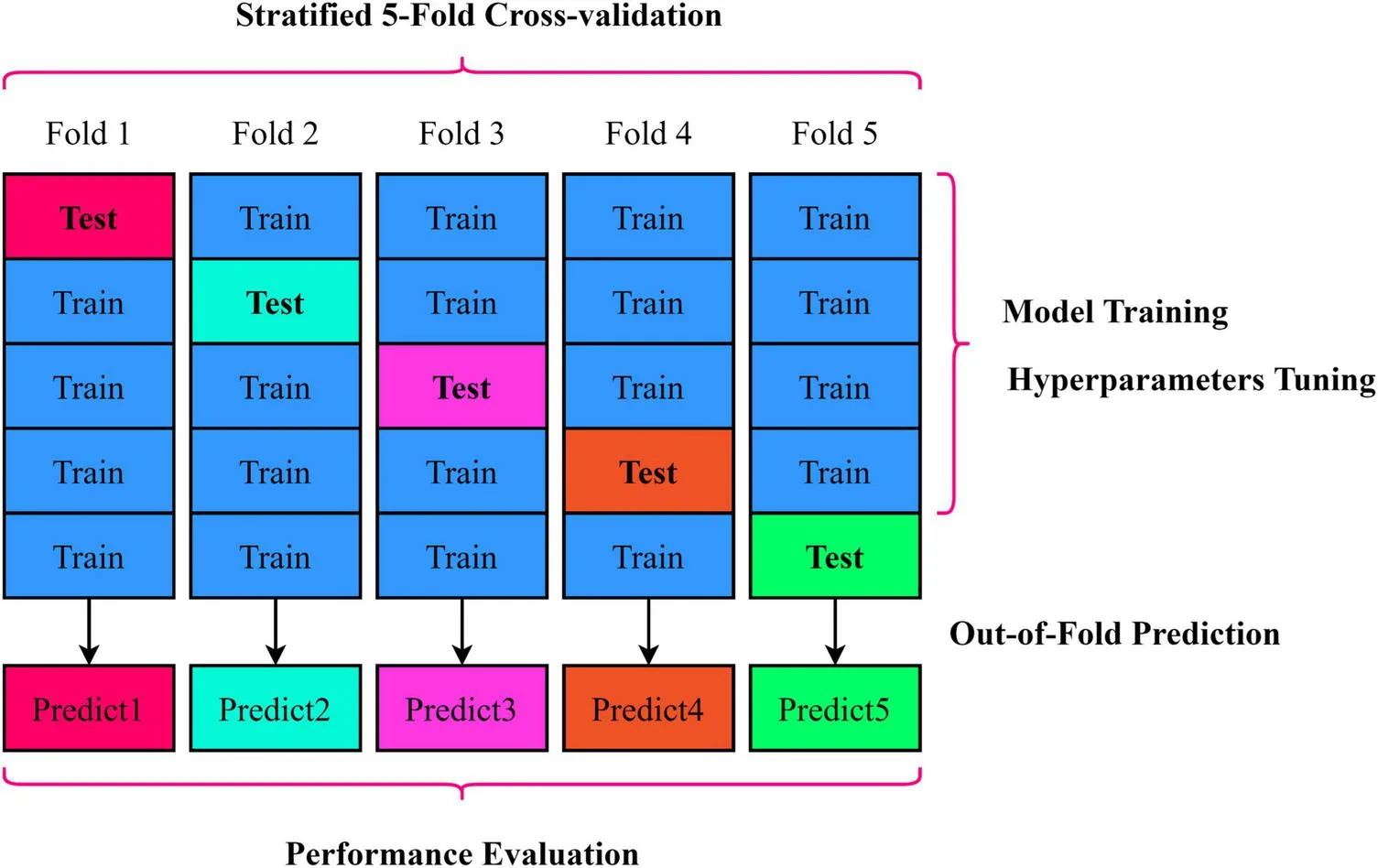
Schematic illustration of the NCV process. It was employed to train the ML model, thereby ensuring the robustness of the training process and the reliability of the evaluation results.

To ensure rigorous validation and minimize performance bias, we implemented a NCV framework. We provide a schematic illustration of this process in Figure 2. The outer loop assessed model generalizability, while the inner loop facilitated hyperparameter optimization via Bayesian optimization using the Optuna framework. Hyperparameter optimization in ML aims to find the hyperparameters that make a ML model perform best on validation datasets (16). To ensure full experimental reproducibility, data partitioning for the cross-validation splits was controlled by a fixed random seed (*n* = 2,023).

The optimization objective was to maximize PR-AUC, a metric selected for its robustness in handling the severe class imbalance of the clinical cohort. Unlike standard ROC-AUC, PR-AUC is highly sensitive to minority-class performance, implicitly forcing the RF algorithm to prioritize mortality detection during tree construction. Within each inner fold of nested cross-validation, 200 independent trials were conducted with no early stopping. All internal algorithmic stochastic processes, including Optuna sampling and model initialization, were controlled by a separate global random seed (42) to ensure full reproducibility. The full pre-defined hyperparameter search space for the RF classifier is provided in Table 2 (e.g., estimator’s ranging from 100 to 600 with step = 10).

**TABLE 2:** Hyperparameter search space and optimization boundaries for the Random Forest model.

Hyperparameter	Description	Search range/type	Role in tuning
*n_estimators*	Total number of decision trees in the forest.	100–600 (Step: 10)	Reduces variance and improves stability.
*max_depth*	Maximum depth of each individual tree.	3–10 (Integer)	Controls model complexity and prevents overfitting.
*ccp_alpha*	Complexity parameter used for Minimal Cost-Complexity Pruning.	0.0–0.02 (Continuous)	Prunes weak branches to enhance generalizability.
*criterion*	The function to measure the quality of a split.	(Gini, Entropy)	Selects the optimal feature for node division.
*min_samples_split*	Minimum number of samples required to split an internal node.	4–8 (Integer)	Constrains tree growth to avoid capturing noise.
*min_samples_leaf*	Minimum number of samples required to be at a leaf node.	1–4 (Integer)	Ensures leaf nodes represent a reliable sample size.
*max_features*	Number of features to consider when looking for the best split.	3–14 (Integer)	Promotes tree diversity within the ensemble.
*max_samples*	Fraction of the dataset used to train each individual tree.	0.60–0.90 (Float)	Implements bootstrap aggregating (bagging) logic.

Following optimization, final models were trained on the comprehensive outer training subsets. Clinical decision thresholds were dynamically determined by maximizing the Youden Index, defined as sensitivity + specificity - 1, based on out-of-fold (OOF) probabilities. Candidate thresholds were evaluated across the aggregated OOF predicted probabilities, and the threshold yielding the maximum Youden Index was selected as the reference cutoff. This threshold was used as a data-driven internal reference to balance sensitivity and specificity, rather than as a definitive clinically optimized decision threshold. Given the low prevalence of in-hospital mortality, alternative clinically driven thresholds, such as cutoffs targeting prespecified sensitivity levels, should be evaluated in future prospective validation studies. Detailed records of the final selected hyperparameters for each fold are provided in Table 3. To ensure consistency in model performance reporting, the primary performance estimates were calculated from aggregated out-of-fold predictions across the five outer validation folds. Specifically, the predicted probabilities from the held-out samples in each outer validation fold were concatenated to form a single aggregated OOF prediction set, from which the performance metrics were then calculated.

**TABLE 3:** Optimal hyperparameter configurations identified for each outer fold of the nested cross-validation.

Model type	Fold	*n_estimators*	*criterion*	*max_depth*	*min_samples_* *split*	*min_samples_ leaf*	*max_features*	*ccp_alpha*	*max_samples*
Full model	1	570	Gini	3	5	4	13	0.0096	0.7225
	2	130	Gini	10	7	4	10	0.0149	0.6832
	3	290	Gini	9	6	4	13	0.0162	0.8842
	4	220	Gini	8	4	2	11	0.0072	0.8882
	5	420	Gini	9	6	1	11	0.0175	0.8844
Parsimonious model	1	290	Gini	9	5	1	7	0.0173	0.7035
	2	550	Gini	6	6	4	5	0.0191	0.8870
	3	260	Gini	9	7	4	6	0.0001	0.8912
	4	200	Gini	8	6	3	6	0.0120	0.8455
	5	580	Gini	6	6	1	7	0.0136	0.8964

### Model interpretation

To provide insights into the interpretability of model predictions, we employed SHAP values to assess the influence of individual predictors on the clinical outcome. SHAP analysis enables the quantification of each feature’s importance in the model’s decision-making process, facilitating explanations of the model’s predictive behavior. SHAP is an effective method to address the interpretability of ML models, which belongs to the method of *post hoc* explanation approach characterized by consistent personalized feature attribution. The central concept is the marginal gain of an individual in a cooperative game. The importance of an individual is determined by calculating its contribution to the collaboration. This is determined by calculating the return of a feature within a feature portfolio, subtracting the return when the feature is absent, and then computing the weighted average across all portfolios to ascertain the overall contribution of the feature, considering all features as “contributors”. For each prediction sample, the model produces a predicted value, and the Shapley value represents the contribution assigned to each feature within that sample. Consider the *i*th sample as *x*_*i*_, the *m*th feature of the *i*th sample as *x*_*im*_, the model’s prediction value for that sample as *y*_*i*_, and the model’s baseline (typically the mean of the target variable across all samples) as *y*_*base*_. The SHAP value is then derived using the following equation:


$$Y_{i}=Y_{b\,a\,s\,e}+f\,\left(x_{i\,1}\right)+f\,\left(x_{i\,2}\right)+\cdots +f\,\left(x_{i\,m}\right)$$ (1)

Within the equation, (*f*x_*im*_) denotes the SHAP value corresponding to the feature *x*_*im*_. Intuitively, (*f*x_*i1*_) represents the contribution of the first feature in the *i*th sample towards the final prediction value *y*_*i*_. If f(x_*i1*_) > 0,it signifies that this feature exerts a positive influence on the prediction, thereby improving the prediction value. Conversely, if f(x_*i1*_) < 0, it implies that the feature negatively influences the prediction, consequently reducing the prediction value.

### Statistical analysis

Continuous variables were compared using two independent sample *t*-test or Mann–Whitney U test based on timing of surgery. Categorical variables were compared with χ2 test or Fisher exact test, depending on the sample size. *P*-value < 0.05 was considered statistically significant.

## Results

### Basic characteristic of clinical samples

Based on the enrollment criteria, the final study cohort included 639 patients. The overall in-hospital mortality rate was 5.6% (36/639). Baseline demographic and comorbidity characteristics are shown in Table 4. No baseline demographic or comorbidity variable differed significantly between survivors and non-survivors (all *p* > 0.05); hypertension showed a non-significant trend toward higher prevalence among non-survivors (83.3% vs. 68.3%, *p* = 0.058).

**TABLE 4:** Baseline demographic characteristic.

Variable	Survivors (*N* = 603)	Non-survivors (N = 36)	*P*-value
Male, n (%)	401 (66.50%)	19 (52.78%)	0.0919
Age, median [Q1, Q3]	54.00 [46.00, 64.00]	57.00 [48.75, 65.00]	0.2650
Hypertension, n (%)	412 (68.33%)	30 (83.33%)	0.0582
Diabetes, n (%)	12 (1.99%)	2 (5.56%)	0.1837
Coronary heart disease, n (%)	58 (9.62%)	6 (16.67%)	0.1601
Cerebrovascular disease, n (%)	71 (11.77%)	7 (19.44%)	0.1867
Marfan syndrome, n (%)	6 (1.00%)	0 (0.00%)	1.0000
Previous cardiac surgery, n (%)	15 (2.49%)	1 (2.78%)	0.6091
Pericardial tamponade, n (%)	14 (2.32%)	0 (0.00%)	1.0000
Coronary involvement, n (%)	125 (20.73%)	9 (25.00%)	0.5409
Limb involvement, n (%)	78 (13.15%)	4 (11.43%)	1.0000
Abdominal organ involvement, n (%)	16 (2.65%)	0 (0.00%)	1.0000
Renal involvement, n (%)	46 (7.63%)	3 (8.33%)	0.7505

Laboratory results upon admission revealed that non-survivors had significantly higher levels of aspartate aminotransferase (AST), myoglobin, creatine kinase-MB (CK-MB), fibrinogen degradation products (FDP), along with significantly lower levels of fibrinogen and eosinophil percentage and count, compared with survivors. All other laboratory variables did not differ significantly between the two groups.

### Our model is a good predictor of in-hospital mortality risk

**FIGURE 3:**
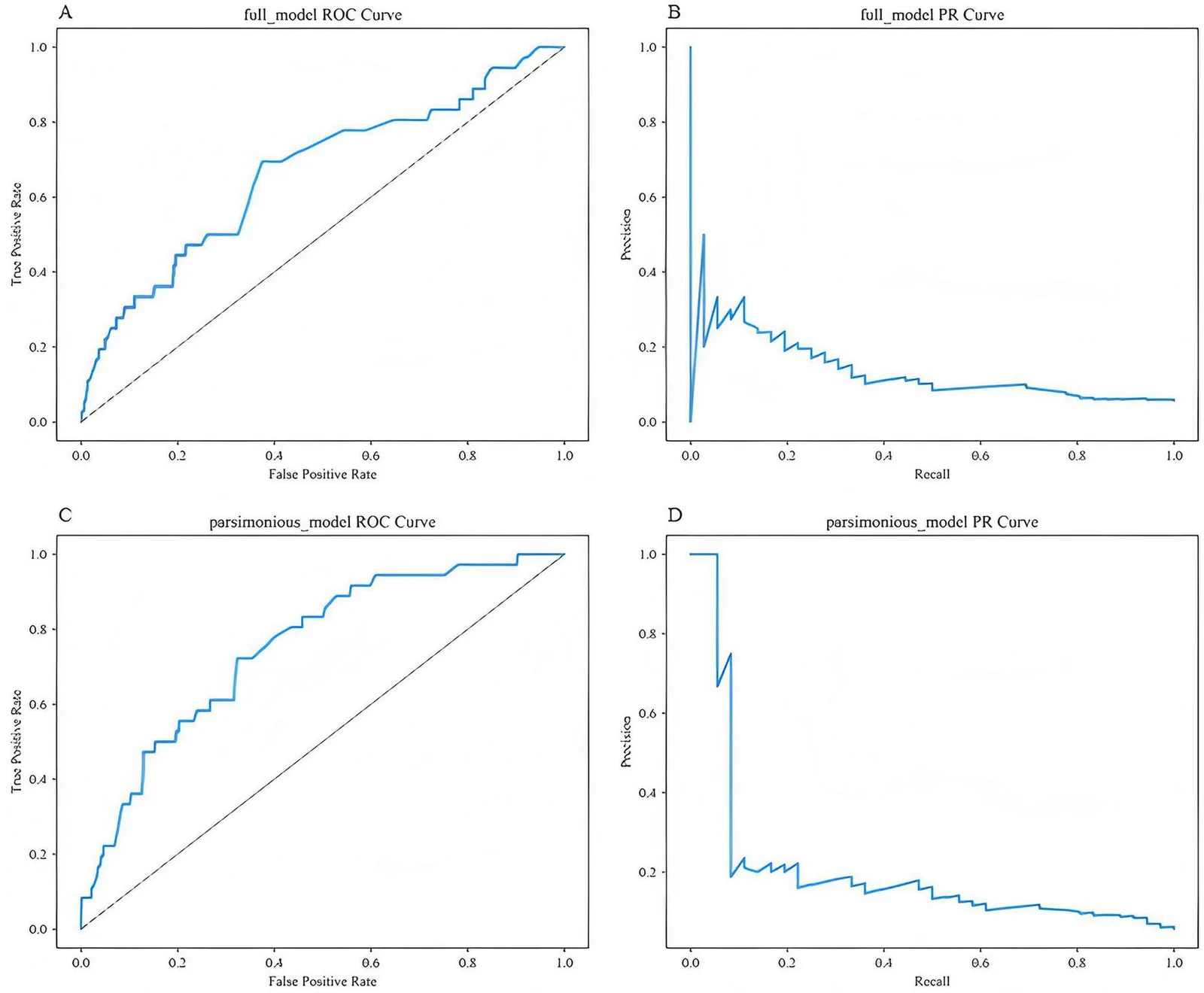
Comparative performance of the prediction models. **(A,B)** the ROC and precision-recall (PR) curves for the full-variable model, respectively; **(C,D)** the ROC and PR curves for the parsimonious model, respectively. The curves were generated using aggregated out-of-fold predictions from the five outer validation folds. The full-variable model achieved an ROC-AUC of 0.666 and a PR-AUC of 0.145, whereas the parsimonious model achieved an ROC-AUC of 0.752 and a PR-AUC of 0.207. ROC-AUC, area under the receiver operating characteristic curve; PR-AUC, area under the precision-recall curve.

The predictive performance was evaluated using NCV ensure the robustness of our clinical findings. Our results demonstrated that the parsimonious model, constructed using the top-ranked prognostic features, significantly outperformed the full-variable model. Specifically, the parsimonious model achieved an AUC of 0.752 and a PR-AUC of 0.207, compared to 0.666 and 0.145 for the full model, respectively (Figure 3).

Given the severe class imbalance in our cohort, we prioritized Sensitivity (Recall) and probability calibration over overall accuracy. Evaluated at the optimized decision threshold (0.0526), the parsimonious model demonstrated high clinical utility as an early-warning triage aid. It achieved a Sensitivity of 0.722, a Specificity of 0.677, and a Balanced Accuracy of 0.701. While the low prevalence of mortality (5.6%) naturally constrained the Positive Predictive Value (PPV) to 0.118, the model achieved an exceptional Negative Predictive Value (NPV) of 0.976. This high NPV enables clinicians to rule out low-risk trajectories, highlighting the model’s superiority as a screening tool to prioritize intensive monitoring.

To address the limitations of traditional goodness-of-fit testing, model calibration was evaluated using Brier scores, calibration intercepts, and slopes derived from aggregated OOF predictions. Within the nested cross-validation framework, we implemented a leakage-safe *post hoc* calibration strategy (Isotonic Regression) to ensure that risk estimates remained unbiased and reliable for early risk stratification.

**FIGURE 4:**
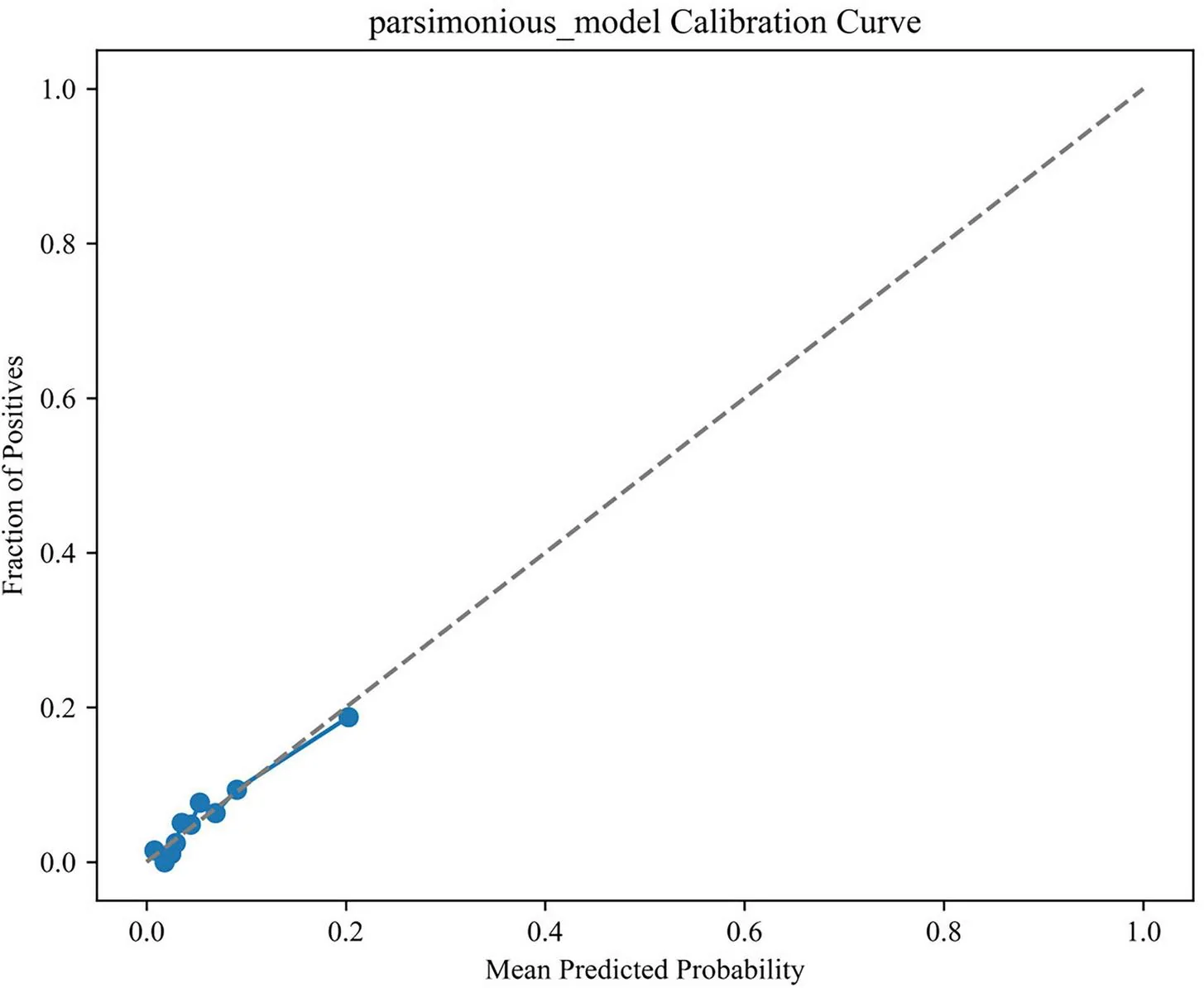
Calibration curve of the parsimonious model for predicting in-hospital mortality. The x-axis represents the mean predicted probability of in-hospital mortality, and the y-axis represents the observed fraction of mortality. The dashed gray line represents the ideal 45-degree calibration where predicted risks perfectly match observed frequencies. The blue dots indicate binned observations, and the solid blue line represents the smoothed calibration trend.

Both models exhibited excellent calibration, with the parsimonious model demonstrating superior performance, as evidenced by a lower Brier score of 0.050, a calibration slope of 0.934, and a calibration intercept of -0.178. These values indicate that the predicted probabilities were robustly aligned with the observed clinical outcomes across the entire risk spectrum (Figure 4). A comprehensive overview of all performance metrics for both the full and parsimonious models, contextualizing both the global calibration and the clinical trade-off between sensitivity and false alarms, is summarized in Table 5.

**TABLE 5:** Comparison of predictive performance and calibration metrics between the full-variable and parsimonious models.

Model type	Fold	roc_auc	pr_auc	Recall/sensitivity	Balanced accuracy	ppv	npv	Brier score	Calibration slope
Full model	1	0.7367	0.2621	0.7143	0.667	0.098	0.974	0.0472	0.8362
	2	0.5992	0.1424	0.5714	0.563	0.069	0.957	0.0504	
	3	0.7066	0.1483	0.7143	0.729	0.139	0.978	0.0506	
	4	0.6542	0.1548	0.75	0.658	0.103	0.971	0.0581	
	5	0.6946	0.1816	0.7143	0.678	0.104	0.975	0.0506	
Parsimonious mdoel	1	0.8436	0.3388	1	0.781	0.117	1	0.0451	0.9343
	2	0.7308	0.1411	0.5714	0.658	0.114	0.968	0.0494	
	3	0.7556	0.2754	0.7143	0.671	0.1	0.974	0.0504	
	4	0.6974	0.2421	0.625	0.692	0.147	0.968	0.0561	
	5	0.8060	0.1752	0.7143	0.703	0.119	0.976	0.0503	

### Identification of key factor for mortality through model interpretation

**FIGURE 5:**
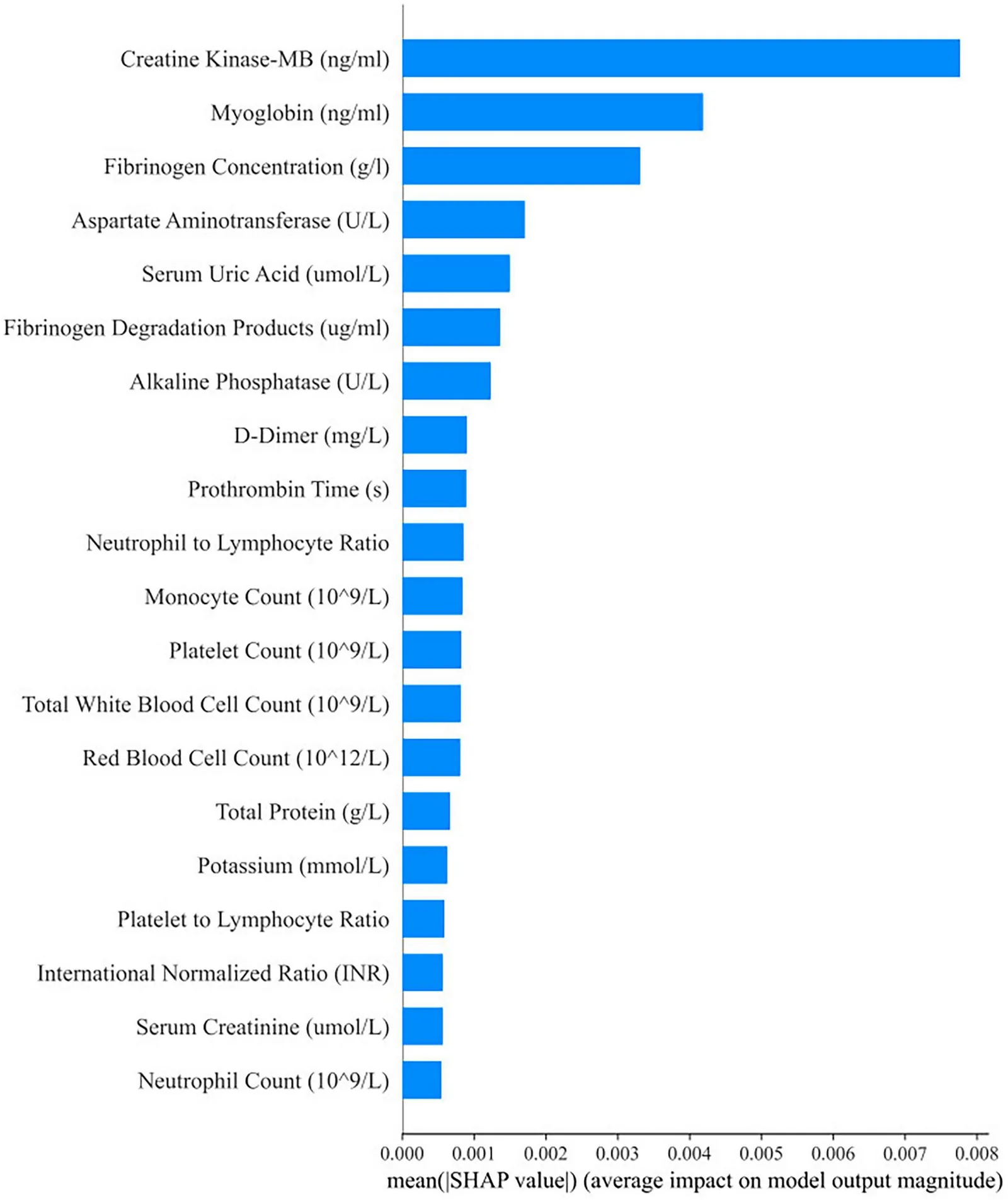
Global feature importance based on mean absolute SHAP values. The bar chart illustrates the overall contribution of preoperative clinical biomarkers to the RF model’s predictions. Features are ranked by their average impact magnitude, defined as mean (SHAP value). CK-MB, Myoglobin, and Fibrinogen Concentration emerge as the top three predictors of in-hospital mortality. This plot quantifies the relative importance of each variable across the entire patient cohort.

**FIGURE 6:**
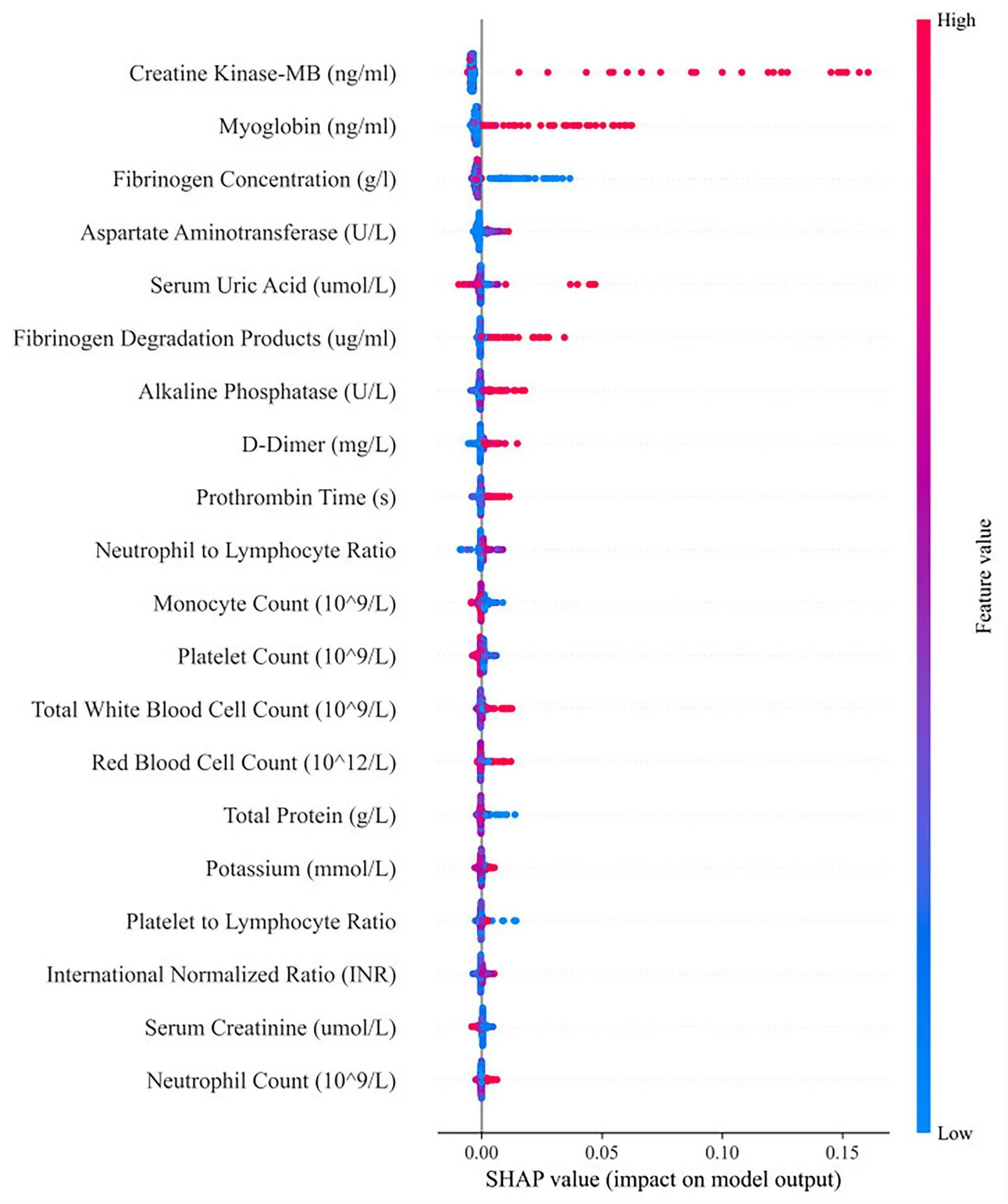
SHAP summary plot illustrating the distribution of feature impacts. This plot combines feature importance with their directional influence on model output. Each point represents an individual patient. The x-axis shows the SHAP value, where positive values indicate an increased risk of mortality. The color gradient represents the feature value (red for high, blue for low).

In relation to the RF model, Figure 5 presents a bar chart illustrating feature importance, aiding in the identification of various clinical features’ significance in the development of the final predictive model. The four highest-ranked selected features (by mean impurity-based importance) were CK-MB, Myoglobin, Fibrinogen Concentration, and Serum Uric Acid (Table 6). These features are key to forming model predictions and to understanding the determinants of risk of in-ospital mortality. For interpretation, global SHAP ranking (Figures 5, 6) additionally highlights AST among the leading contributors. We distinguish the two throughout: feature selection for the parsimonious model used mean impurity importance, whereas feature interpretation used mean absolute SHAP values.

**TABLE 6:** New (15 parsimonious features).

Rank	Feature	Unit	Mean importance
1	Creatine kinase-MB (CK-MB)	ng/mL	0.2243
2	Myoglobin	ng/mL	0.0871
3	Fibrinogen concentration	g/L	0.0702
4	Serum uric acid	μmol/L	0.0583
5	Fibrinogen degradation products (FDP)	μg/mL	0.0368
6	Alkaline phosphatase (ALP)	U/L	0.0317
7	Potassium	mmol/L	0.0309
8	D-Dimer	mg/L	0.0279
9	Platelet-to-lymphocyte ratio (PLR)	Dimensionless	0.0268
10	Sodium	mmol/L	0.0267
11	Prothrombin time (PT)	s	0.0265
12	Total protein	g/L	0.0229
13	Neutrophil count	10^9^/L	0.0228
14	International normalized ratio (INR)	Dimensionless	0.0218
15	γ-glutamyl transpeptidase (GGT)	U/L	0.0211

SHAP’s foremost advantage is used to reflect the influence of each feature in every sample, providing local-level quantitative explanation beyond mere feature importance, and also illustrating the direction of this influence. Figure 6 exhibits the SHAP values of the 20 most important features in the constructed RF model. The biomarkers are ranked by their overall importance to the model, with CK-MB having the highest impact and Neutrophil Count having the lowest. CK-MB and Myoglobin are demonstrated to be the strongest predictors, consistent with their role as markers of potential myocardium and extremity damage. The complex patterns observed for some biomarkers, such as Fibrinogen and Serum Uric Acid, suggest non-linear relationships between these markers and the condition being predicted. This analysis highlights the potential of ML approaches in integrating multiple biomarkers to improve diagnostic or prognostic accuracy in clinical settings.

In terms of global interpretability, the analysis yields the top 15 features affecting the model’s predictions. An increase in a feature’s SHAP value correlates positively with an elevated risk of in-hospital mortality. Within the individual patient model, each feature’s attribution value is depicted in a dot format, assigning respective point values to each patient for different features. These dots are color-coded based on the patient’s feature values and accumulate vertically to illustrate the distribution of feature value density. This method elucidates how each feature impacts the final outcome, either increasing or decreasing the prediction value relative to a baseline prediction. The schematic in Figure 6 clearly demonstrates this process. To further elucidate the complex decision-making logic, we generated SHAP dependence plots for top-ranking predictors, which visualize how specific clinical values translate into mortality risk.

**FIGURE 7:**
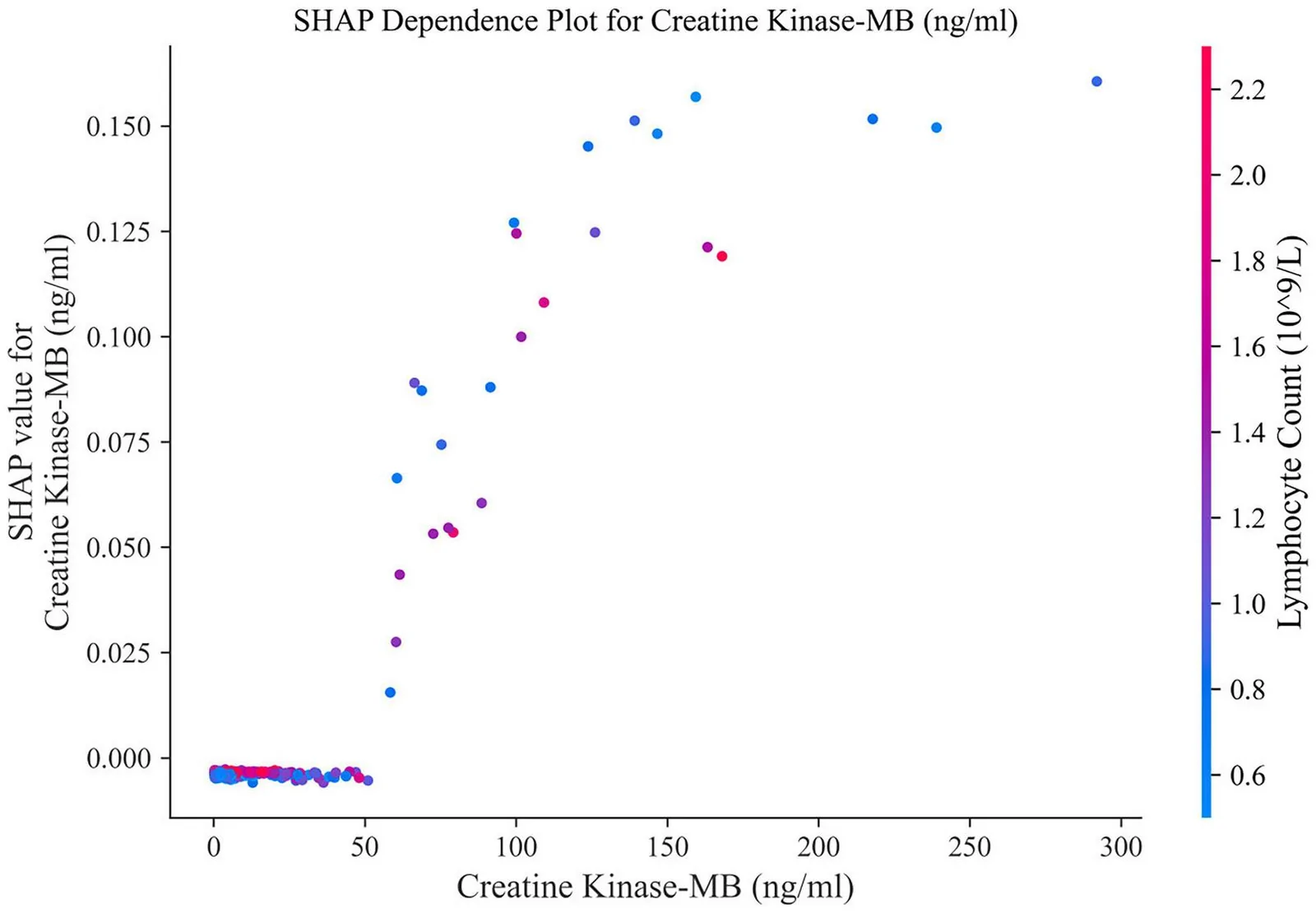
SHAP dependence plot for CK-MB. The x-axis shows CK-MB levels (ng/mL), and the y-axis shows the corresponding SHAP values. Higher CK-MB levels, particularly above approximately 50 ng/mL, were associated with higher model-predicted mortality risk. Point color represents lymphocyte count.

As illustrated in Figure 7 (CK-MB), a distinct risk-transition threshold was identified at approximately 50 ng/ml. Below this level, CK-MB exerts a minimal or slightly protective influence on the model’s output; however, beyond this threshold, the SHAP value escalates sharply and monotonically, confirming that myocardial injury is a primary driver of adverse outcomes. The color-coded interaction with Lymphocyte Count further suggests that the prognostic impact of cardiac damage is modulated by the patient’s systemic inflammatory state.

**FIGURE 8:**
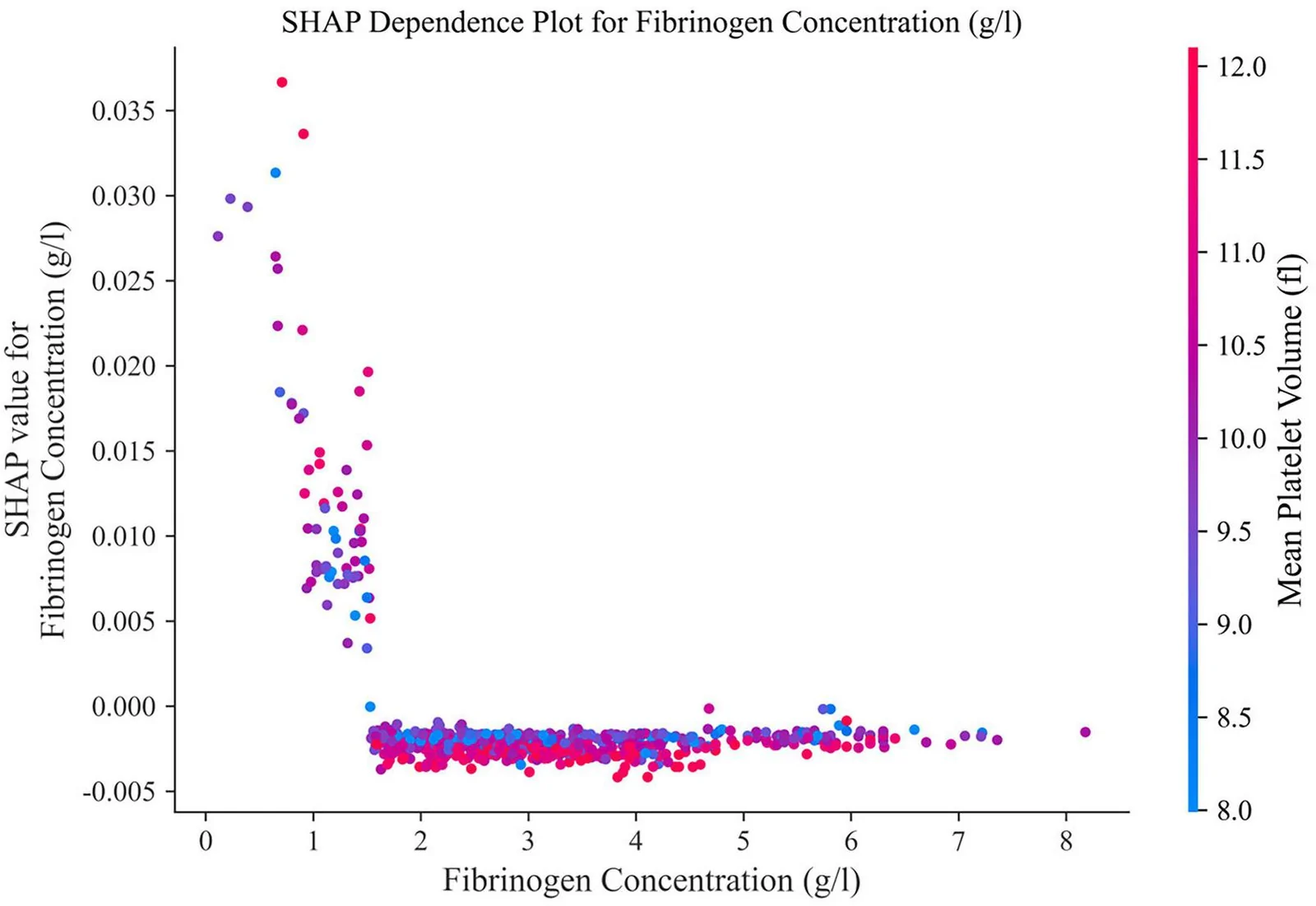
SHAP dependence plot for Fibrinogen Concentration. The plot characterizes the relationship between preoperative fibrinogen levels and model output. An inverse relationship is evident: as fibrinogen levels drop below 1.5 g/l, the SHAP values increase substantially, identifying hypofibrinogenemia as a significant driver of mortality risk, likely reflecting acute consumptive coagulopathy in ATAAD patients. The color bar indicates the interaction with MPV. The plateau observed above 2.0 g/l suggests that the incremental protective effect of higher fibrinogen levels diminishes beyond this range.

Similarly, Figure 8 (Fibrinogen Concentration) reveals a critical non-linear relationship where the risk of in-hospital mortality surges as fibrinogen levels drop below 1.5 g/l. This pattern captures the “consumptive coagulopathy” typical of high-risk ATAAD patients. The interaction with Mean Platelet Volume (MPV) indicated by the vertical color variance highlights the synergistic effect between coagulation factor depletion and platelet activation in predicting fatal events. Importantly, the consistency of the risk trend even at extreme pathological levels (e.g., CK-MB > 250 ng/ml or Fibrinogen < 1.0 g/l) validates that the model captures authentic biological signals rather than statistical artifacts.

**FIGURE 9:**
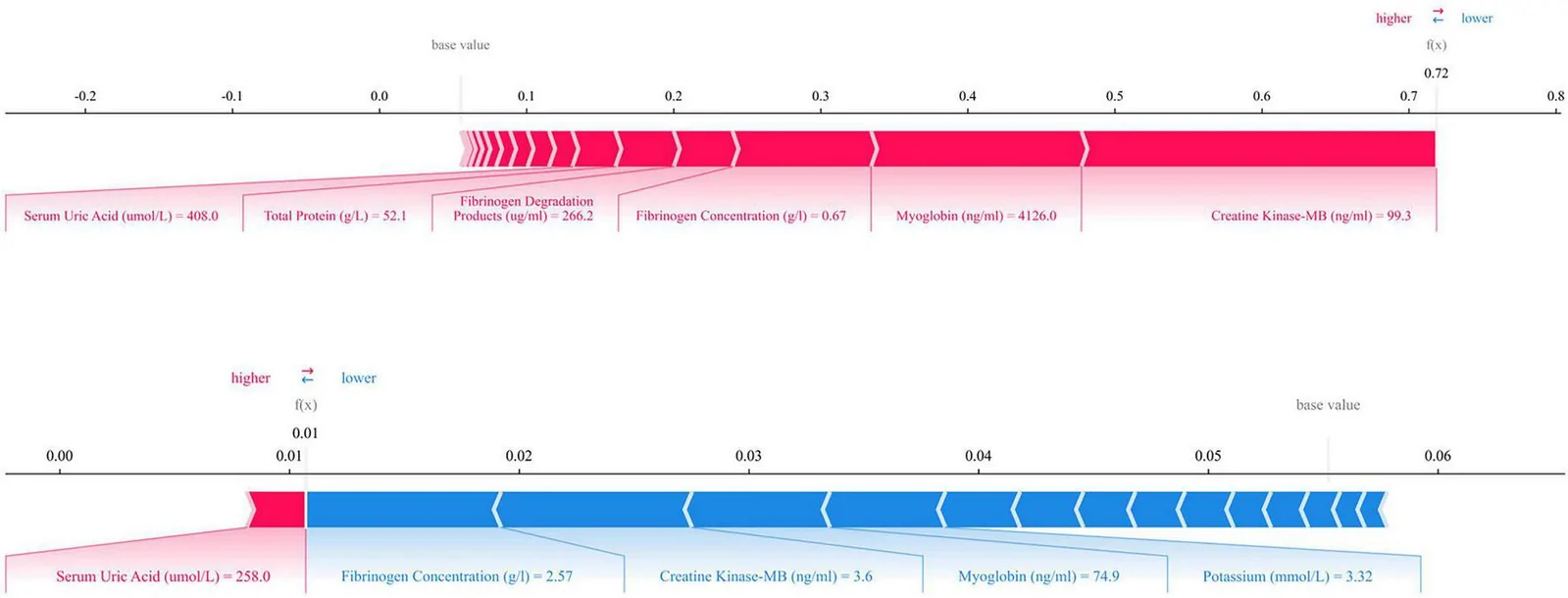
SHAP force plots demonstrate the influence of various biomarkers on model predictions for two distinct cases. For individual prediction instance, the SHAP value elaborates on how each feature impacts the model output, specifically illustrating the shift from the baseline value to the current prediction.

As shown in Figure 9, the SHAP force plots illustrate individualized risk predictions using the parsimonious model. To accurately reflect real-world clinical scenarios, the baseline (expected) value was mathematically calibrated to 0.056, aligning with the 5.6% in-hospital mortality prevalence observed in our cohort. The SHAP force plots delineate a consistent high- versus low-risk profile. High-risk patients were typically characterized by elevated CK-MB and myoglobin, hypofibrinogenemia, and elevated FDP—a constellation attributable to the three principal pathophysiologic mechanisms of ATAAD: coronary/myocardial involvement (CK-MB), systemic hypoperfusion and tissue injury (myoglobin), and consumptive coagulopathy with fibrinolytic activation (low fibrinogen, high FDP)—whereas low-risk patients showed values within comparatively safe ranges. The two representative cases below illustrate this pattern at the individual level.

In the first case (top plot), the model predicts a mortality probability of 0.72, substantially higher than baseline. This extreme risk was primarily driven by markedly elevated FDP (266.2 μg/mL), Myoglobin (4,126 ng/mL), and CK-MB (99.3 ng/mL), together with a critically reduced Fibrinogen Concentration (0.67 g/L). These biomarkers are strongly associated with profound coagulopathy, severe muscle injury, and cardiac events. The extreme values identified in Case 1 (e.g., FDP 266.2 μg/mL) were not statistical artifacts; outlier diagnostics revealed that 11.58% of patients in our cohort exhibited FDP levels > 100 μg/ml, consistent with systemic fibrinolysis commonly observed in severe ATAAD.

In the second case (bottom plot), the model predicts a mortality probability of 0.01, classifying the patient as low risk. A slightly elevated Serum Uric Acid (258.0 μ mol/L) exerted a minor upward influence, but this was strongly counterbalanced by protective factors (blue arrows) that reduced the risk well below baseline. These protective states included normal or non-critical levels of Potassium (3.32 mmol/L), Myoglobin (74.9 ng/ml), CK-MB (3.6 ng/ml), and preserved Fibrinogen Concentration (2.57 g/l). Consistent with global feature importance analysis (Figure 5), severe tissue hypoperfusion (high CK-MB/Myoglobin) and consumptive coagulopathy (low Fibrinogen Concentration) are strong indicators of elevated mortality risk, whereas values within safer ranges effectively mitigate risk.

These graphical representations elucidate the complex interplay among biomarkers in predicting clinical outcomes, underscoring the importance of multifactorial analyses in medical decision-making. The model’s ability to transparently integrate competing physiological factors across diverse clinical scenarios underscores its potential as a personalized triage tool in practice.

## Discussion

In the present study, we established and validated a ML risk prediction model for ATAAD surgical outcomes, in which we selected the RF algorithm as the primary method. As shown in the results, artificial intelligence (AI) has significant advantages for predicting events based on RF algorithms. Multiple ML techniques have also shown significant features in predicting in-hospital mortality with the enhanced performance and incorporating more variables.

ATAAD is a catastrophic condition with high surgical mortality and morbidity. Despite remarkable improvements in surgical techniques and perioperative management, the surgical mortality in ATAAD surgery remains high. How to identify risk factors for in-hospital mortality will provide more information on clinical practices and shared clinical decision making. Currently there are already well-established and traditional prediction models for cardiac surgery mortality prediction, including EuroSCORE II and German Registry of Acute Aortic Dissection Type A (GERAADA). However these models are only not intended for ATAAD patients and lack of specificity for ATAAD patients. Ma and colleagues reported their study in comparing the two scoring systems about ATAAD surgical mortality prediction. In their retrospective study of 1346 ATAAD patients, the observed surgical mortality was 12.5%, while the GERAADA score and EuroSCORE II predicted 30-day mortality rates were 14.7 and 3.1%, respectively (17). Due to the complexity and nature of aortic surgery, a more acute and specific prediction model is urgently needed.

We did not perform a head-to-head comparison with EuroSCORE II or GERAADA because the current dataset does not contain all variables required to compute these scores with their published definitions; we did not impute score components. The proposed model uses admission biomarkers only and is intended to complement, not replace, established surgical risk scores as an early biomarker-based triage layer.

In recent years, the application of AI in aortic dissection has gained more attention. A recent study used logistic regression and AI algorithms to examine risk factors for the early rupture of ATAAD, which could improve the performance of early precision diagnostic and prognostic outcome evaluation. Besides, the AI approaches outperformed conventional models in terms of sensitivity, specificity, and prediction accuracy (18). Furthermore, in a recent study by Xie, they utilized six different ML algorithms to predict the incidences of postoperative adverse outcomes after total arch replacement in treating ATAAD in a cohort of 380 patients. They compared the performances and outcomes of different algorithms and showed that the eXtreme Gradient Boosting (XGBoost) model is the optimal model (19). However, the precision and accuracy of AI in prediction surgical mortality with variables on admission remains unclear. Thus in our study, we aim to develop and validate ML- based models to precisely predict in-hospital mortality in surgical patients with ATAAD. There are several strengths of our study. Firstly, we adopted variables at admission of the patients. The onset of ATAAD is always emergent, so early and quick recognition in stratifying high-risk patients is always crucial in the clinical setting. Secondly, we incorporated multiple laboratory values, of which could be recognized as significant features. The SHAP model method was introduced in our study, for more clear description of the significant features. Thirdly, the ML-based modes in our study had certain interpretability. They show flexibility to capture complex non-linear relationships with missing data.

Among the significant features, we found that the CK-MB is the most significant feature in our model. ATAAD with CK-MB elevation was found in patients with coronary artery involvement, which was found significantly associated with higher surgical mortality (20). This is in consistency with our finding. Patients with acute coronary artery and even acute myocardial infarction are more prone to experience cardiac dysfunction and low cardiac output syndrome during hospitalization, which is highly associated with in-hospital mortality. Apart from CK-MB, myoglobin was recognized as the second significant feature. Myoglobin is reported to be a predominant predictor of acute kidney injury and more sensitive than serum creatine in ATAAD surgery, which would undoubtedly increase mortality (21). The significant features illustrated with ML techniques are of total consistency with clinical true scenario. From a model-selection perspective, the parsimonious 15-variable model should be prioritized over the full-variable model for future validation and potential clinical translation. First, ATAAD is an emergency condition with a relatively low number of mortality events available for model development in most single-center cohorts. In this context, reducing the number of predictors is methodologically important because a lower-dimensional model may be less vulnerable to instability and overfitting than a full-variable model. Second, a smaller predictor set is more feasible for rapid presurgical risk assessment and easier to reproduce across different clinical cohorts, especially when laboratory availability, assay platforms, and data completeness vary between centers. Third, in our internal validation, the parsimonious model showed numerically better discriminatory performance than the full-variable model, with a higher ROC-AUC (0.752 vs. 0.666) while using substantially fewer predictors. Therefore, the full model should be interpreted mainly as an exploratory reference model, whereas the parsimonious 15-variable model represents the preferred candidate for external validation and potential admission-based risk stratification.

Given a mortality prevalence of 5.6%, the PPV of 0.118 means that a positive result cannot confirm mortality risk, whereas the NPV of 0.976 supports the model’s primary role as an admission rule-out/screening layer. The Youden-derived threshold of 0.0526 should be interpreted as an internally derived reference cutoff rather than a definitive clinical decision threshold. Although this threshold provided a data-driven balance between sensitivity and specificity, it does not directly account for event prevalence, resource availability, or the unequal clinical consequences of false-negative and false-positive classifications. A negative classification identifies patients at relatively low observed risk; a positive classification should prompt intensified monitoring, repeated hemodynamic and laboratory reassessment, and heightened perioperative vigilance, but should not by itself alter surgical indication, delay surgery, or justify treatment limitation. Given the specificity of 0.677, approximately 32.3% of survivors would be expected to be classified as positive, indicating a considerable false-positive burden. Therefore, the model should be viewed as a preliminary early-warning and rule-out tool rather than a definitive mortality prediction tool. Future multicenter prospective studies should evaluate alternative clinically driven thresholds, including thresholds targeting higher prespecified sensitivity, before any routine clinical implementation is considered. Because NPV depends on prevalence, this role requires external validation in centers with different baseline risk.

Some limitations should be mentioned in our study. Firstly, the study was limited by the nature of the retrospective study, as all the date were retrospectively collected from the database. Secondly, there was some missing data in the early phase patients in our study. Thirdly, external validation is lack in our study while necessitates more external data to validate the accuracy of the model. Lastly, as the actual surgical outcome can be interfered by diverse intraoperative and postoperative factors, a system with more intraoperative and postoperative can be established in the future.

In conclusion, a model for prediction of in-hospital mortality after ATAAD surgical treatment has been established using ML techniques. This tool can provide relevant information in clinical practice and to assist in the decision-making process.

## Limitations

The main limitation of this study is its limited sample size, which reflects the single-center retrospective design. The number of in-hospital deaths was small, resulting in a low events-per-variable ratio and a potential risk of model instability or overfitting, particularly for the full-variable model. Although nested cross-validation, fold-wise imputation, probability calibration, and threshold optimization were used to reduce optimism and data leakage, these procedures cannot substitute for external validation. Therefore, further multicenter external validation is warranted to assess the transportability, calibration, and clinical utility of the proposed model.

Another limitation is that we did not perform a formal head-to-head comparison with penalized logistic regression, such as LASSO-logistic regression, or with other machine-learning algorithms, including XGBoost and support vector machine, under the same nested validation framework. Accordingly, we make no claim that the RF-based model is superior to these alternative approaches. Although RF was selected because it can capture non-linear associations and potential feature interactions, does not require predictor scaling, and can be integrated with fold-wise imputation, probability calibration, and SHAP-based interpretation, this selection was based on methodological suitability rather than demonstrated empirical superiority in the present cohort. Therefore, the current study cannot determine whether RF represents the optimal algorithm for predicting in-hospital mortality after ATAAD surgery. Future studies should compare multiple candidate algorithms within a unified validation framework to determine the most appropriate modeling strategy for ATAAD risk prediction.

Transportability may be limited by between-center differences in laboratory assay platforms, reagent lots, reference intervals and calibration; in surgical indications, repair strategy, perioperative protocols and ICU resources; and in age, comorbidity and disease-severity case-mix. We propose a multicenter, prospective validation that locks the model and threshold, reports center-specific and pooled ROC-AUC/PR-AUC/calibration and net benefit, performs recalibration only if needed, evaluates alert burden during a silent-run phase, and considers decision-support integration only after a prospective impact study.

## Conclusion

The combined RF model effectively addresses the imbalanced dataset and improves the reliability of ATAAD mortality predictions. While the false-positive burden necessitates cautious application, this model serves as a highly valuable triage and screening aid in clinical practice, facilitating early risk stratification and optimizing resource allocation for high-risk patients.

## Data Availability

The raw data supporting the conclusions of this article will be made available by the authors, without undue reservation.
